# Clinical, Epidemiologic, and Pathologic Significance of ERBB2-Low Expression in Breast Cancer

**DOI:** 10.1001/jamanetworkopen.2024.3345

**Published:** 2024-03-22

**Authors:** Thaer Khoury, Lucas Mendicino, Rochelle Payne Ondracek, Song Yao, Warren Davis, Angela R. Omilian, Marilyn L. Kwan, Janise M. Roh, Lia D’Addario, Emily Valice, Daniel Fernandez, Isaac J. Ergas, Alfredo V. Chua, Christine B. Ambrosone, Lawrence H. Kushi

**Affiliations:** 1 Department of Pathology, Roswell Park Comprehensive Cancer Center, Buffalo, New York; 2 Department of Cancer Prevention and Control, Roswell Park Comprehensive Cancer Center, Buffalo, New York; 3 Division of Research, Kaiser Permanente Northern California, Oakland

## Abstract

**Importance:**

It is unclear whether breast cancer (BC) with low ERBB2 expression (ERBB2-low) is a distinct clinical, pathological, and epidemiological entity from BC classified as no ERBB2 expression (ERBB2-negative).

**Objective:**

To evaluate the clinical, pathological, and epidemiologic features of BC with ERBB2-low expression compared with ERBB2-negative BC in a large population study.

**Design, Setting, and Participants:**

This cohort study was conducted as part of the Pathways Study, a prospective, racially and ethnically diverse cohort study of women with BC enrolled between 2006 and 2013 in Kaiser Permanente Northern California (KPNC). The hematoxylin and eosin slides underwent centralized pathology review, including the percentage of tumor infiltrating lymphocytes (TILs). Breast biomarker results were extracted from pathology reports, and women were included if they had a documented ERBB2 value that was not classified ERBB2-positive. Data were analyzed from February 2023 through January 2024.

**Exposure:**

Clinical and tumor characteristics associated with BC and ERBB2-low or ERBB2-negative status.

**Main Outcome and Measures:**

ERBB2-low was defined as immunohistochemistry score of 1+ or 2+ (negative by in situ hybridization); ERBB2-negative was defined as immunohistochemistry score of 0+. Other data were collected by self-report or extraction from electronic health records, including BC risk factors, tumor characteristics, treatment modality, and survival outcomes, with recurrence-free survival (RFS) as the primary outcome and overall survival (OS) and BC-specific mortality (BCSM) as secondary outcomes. The clinical, pathological, and epidemiological variables were compared between ERBB2-low and ERBB2-negative BC.

**Results:**

Of 2200 eligible patients (all female; with mean [SD] age, 60.4 [11.9] years), 1295 (57.2%) had tumors that were ERBB2-low. Hormone receptors were positive in 1956 patients (88.9%). The sample included 291 Asian patients (13.2%), 166 Black patients (7.5%), 253 Hispanic patients (11.5%), 1439 White patients (65.4%), and 51 patients (2.3%) who identified as other race or ethnicity (eg, American Indian or Alaska Native and Pacific Islander). Within the hormone receptor–negative group, patients whose tumors had ERBB2-low staining, compared with those with ERBB2-negative tumors, had better OS (hazard ratio [HR], 0.54; 95% CI, 0.33-0.91; *P* = .02), RFS (HR, 0.53; 95% CI, 0.30-0.95; *P* = .03), and BCSM (HR, 0.43; 95% CI, 0.22-0.84; *P* = .01). In multivariable survival analysis stratified by hormone receptor status and adjusted for key covariates, patients with ERBB2-low and hormone receptor–negative tumors had lower overall mortality (HR, 0.48; 95% CI, 0.27-0.83; *P* = .009), RFS (HR, 0.45; 95% CI, 0.24-0.86; *P* = .02), and BCSM (subdistribution HR, 0.21; 95% CI, 0.10-0.46; *P* < .001) compared with patients with ERBB2-negative and hormone receptor–negative tumors. Within the hormone receptor–negative subtype, patients with ERBB2-low and high TILs tumors had better survival across all 3 outcomes compared with patients with ERBB2-negative and low TILs tumors. Additionally, patients with ERBB2-low and low TILs tumors had better BCSM (subdistribution HR, 0.36; 95% CI, 0.14-0.92; *P* = .03).

**Conclusions and Relevance:**

These findings suggest that there were clinical, pathological, and epidemiological differences between ERBB2-low and ERBB2-negative BC, raising the possibility that ERBB2-low might be a unique biologic entity.

## Key Points

**Question:**

Is ERBB2-low breast cancer a distinct biologic entity?

**Findings:**

In this cohort study of 2200 patients, there were differences in hormone-receptor status, family history, and self-identified race and ethnicity between ERBB2-low and ERBB2-negative status. Within hormone receptor–negative tumors, ERBB2-low status was associated with better survival compared with ERBB2-negative status.

**Meaning:**

These findings suggest that ERBB2-low breast cancer might be a distinct biologic entity.

## Teaser

This cohort study evaluates the clinical, pathological, and epidemiologic features of breast cancer with ERBB2-low expression compared with BC classified as no ERBB2 expression (ERBB2-negative) in a large population study

## Introduction

The human epidermal growth factor receptor 2 (*ERBB2*) gene is amplified relatively frequently in breast cancer (BC), and is associated with disease relapse and patient survival.^1^ Ever since anti-ERBB2 targeted therapy was approved for the treatment of patients with BC, clinical evaluation of ERBB2 has become standard of care and is routinely assessed using immunohistochemistry (IHC) and in situ hybridization (ISH). Patients with ERBB2-positive tumors (scores of 3+ by IHC or *ERBB2* gene amplified by ISH) are eligible for this treatment. The remaining tumors were classified as negative.^2^ However, the published results of the DESTINY-Breast04 phase 3 trial demonstrated the efficacy of anti-ERBB2 antibody-drug conjugate trastuzumab-deruxtecan in patients with ERBB2-low metastatic BC. Therefore, the dichotomous classification (positive vs negative) was challenged, as this new therapy only treats ERBB2-low tumors.^3^ ERBB2-low is defined as 1+ or 2 + by IHC or ISH-negative and was adopted by the new American Society of Clinical Oncology-College of American pathologists (ASCO-CAP) guidelines. However, the guidelines stated that it is premature to change reporting terminology for lower levels of ERBB2 IHC expression (ie, ERBB2-low). ASCO-CAP iterated that there is no evidence that ERBB2-low is a new or reproducibly defined subtype of BC with distinct prognostic implications.^4^

Accumulating evidence from several studies indicates that tumor infiltrating lymphocytes (TILs) are associated with better survival in triple-negative breast cancer (TNBC) and to a lesser extent in ERBB2-positive BC, with fewer studies supporting the relevance of TILs in luminal (hormone receptor–positive and ERBB2-negative) BC.^5,6^ However, the association between TILs and ERBB2 at low levels (ERBB2-negative or ERBB2-low) is unknown.

Although there have been a plethora of publications discussing the clinical and pathologic features of ERBB2-low, more studies are needed, particularly those with prospective evaluation to decrease survival bias and resolve conflicting results. Moreover, the association between ERBB2-low BC and epidemiological factors has not been fully investigated. In this study, we evaluated the clinical, pathologic, and epidemiologic characteristics in patients with amplified-ERBB2 (ERBB2-low vs ERBB2-negative) tumors.

## Methods

This cohort study was conducted as part of the Pathways Study and approved by the institutional review boards of Kaiser Permanente Northern California (KPNC) and Roswell Park Comprehensive Cancer Center. All participants provided written informed consent, including access to clinical records with pathology records and specimens. This study is reported following the Strengthening the Reporting of Observational Studies in Epidemiology (STROBE) reporting guideline.

### Study Population

The Pathways Study is a prospective racially and ethnically diverse cohort of women who were diagnosed with invasive breast cancer through KPNC between 2005 and 2013.^7^ Eligibility criteria included no previous diagnosis of other invasive cancer, age 21 years or older, and ability to speak English, Spanish, Cantonese, or Mandarin. Women were generally enrolled within 2 months of their diagnosis, with an extensive baseline interview conducted. Data included in these analyses were age at diagnosis, self-identified race and ethnicity (SIRE) (reported as Asian, Black, Hispanic, White, or other [eg, American Indian or Alaska Native and Pacific Islander]), body mass index (BMI; calculated as weight in kilograms divided by height in meters squared), menopausal status, family history, parity, smoking status (never, former, or current), and breast feeding (yes vs no). SIRE was included in analyses as ERBB2 status and associations with outcomes may differ by SIRE. Clinical data included treatment modalities, including chemotherapy, radiation therapy, and hormonal therapy, and American Joint Committee on Cancer staging (I, II, III, IV). Survival outcomes were overall survival (OS), recurrence-free survival (RFS), and BC-specific mortality (BCSM). RFS was defined as first clinically reported recurrence or death due to BC. Follow-up began at date of diagnosis and continued until occurrence of BC event; patients with no event of interest were censored at the time of last outcome ascertainment (December 31, 2021). All study participants received their health care, including standard cancer care, through KPNC.

### Pathology Variables

As part of the Pathways Study, the hematoxylin and eosin slides from each patient were centrally reviewed by a single breast pathologist (T.K.). Several variables were recorded during pathological review. Nottingham grade was scored from 1 to 3, incorporating the tubular formation, nuclear pleomorphism, and mitotic count.^8^ Histologic type was classified following World Health Organization criteria, including invasive carcinoma of no special type (IC-NST) (ductal), lobular, tubular, mucinous, cribriform, papillary, carcinoma with apocrine differentiation, micropapillary, micropapillary or mucinous, metaplastic, acinic cell, adenoid cystic, mucoepidermoid, secretory, tall-cell carcinoma with reverse polarity, neuroendocrine, microinvasive, and mixed. To have qualified for special type, the special type component needed to account for more than 90% of the tumor. To have qualified for mixed type, the special type component needed to account for 10% to 90% of the tumor.^9^ The percentage of ductal carcinoma in situ (DCIS) was also recorded. The percentage of TILs (on a scale from 0% to 100% with 10% increments) was recorded following the international TILs working group 2014. In short, to calculate the percentage of TILs, the stromal component was considered the denominator (area occupied by mononuclear inflammatory cells over total intratumoral stromal area). TILs were evaluated in the vicinity of the tumor with excluding surrounding tissue, normal epithelium, and DCIS.^10^

Status of breast biomarkers, including hormone receptors and ERBB2, were abstracted from pathology reports. The scoring for these markers followed the guidelines which were implemented at the time of diagnosis.^11,12,13^ For estrogen receptor (ER) and progesterone receptor (PR), a cutoff of at least 1% was considered positive. Tumors with unclear designation of the ERBB2 status (IHC score of 0+ or 1+), which were reported as negative, were excluded from the study. Patients with stage IV disease or those with ERBB2-positive tumors were also excluded from the study.

### Statistical Analysis

Clinical and demographic characteristics of the Pathways Study cohort were compared by ERBB2 and hormone receptor status. Continuous variables were presented as mean with SD for normally distributed variables, median with IQR for nonnormally distributed variables, and number and percentage for categorical variables. Welch 2-sample *t* test and Wilcoxon rank sum test were used to compare continuous variables, and Pearson χ^2^ and Fisher exact test were used for categorical variables.

Associations of survival outcomes with ERBB2 status (ERBB2-low vs ERBB2-negative) were assessed using Cox proportional hazards models for OS and RFS and Fine-Gray competing risks models for BCSM with non-BCSM as a competing event. The assumption of proportional hazards was assessed using scaled Schoenfeld residuals, and hormone receptor status violated the proportional hazards assumption. Two models were constructed to assess ERBB2. Model 1 stratified by hormone receptor status and adjusted for stage, chemotherapy, hormonal therapy, surgery, and radiation therapy. Model 2 additionally included an interaction between ERBB2 and hormone receptor strata. Model 1 assumes the association of ERBB2 to be the same across hormone receptor strata, and model 2 assumes the association of ERBB2 is different between hormone receptor strata, allowing for estimates of ERBB2 in hormone receptor–positive and hormone receptor–negative subgroups. Subgroup analyses assessed ERBB2 within SIRE groups using an age-adjusted model and model 1. Furthermore, the association of TILs was assessed in 2 ways. First, TILs were modeled continuously to reflect a 10% increase in TILs within ERBB2 and ERBB2 and hormone receptor subgroups using an age-adjusted model and model 1. Then, a 30% cutoff was used to define low (≤30%) vs high (>30%) TILs. A composite variable with ERBB2 and TILs was created to reflect 4 groups (ERBB2-low with high TILs, ERBB2-low with low TILs, ERBB2-negative with high TILs, and ERBB2-negative with low TILs) and its association was assessed using model 1 and model 2. Two-sided *P* = .05 was considered statistically significant without adjustment for multiple comparison, given the exploratory nature of the analysis, and all analyses were conducted in programming language R version 4.2.0 (R Project for Statistical Computing). Data were analyzed from February 2023 through January 2024.

## Results

A total of 4504 patients were in the Pathways Study. Complete pathologic data and definitive ERBB2 designation were available for 2774 patients (61.6%); of those, 545 patients with ERBB2-positive tumors, and 29 patients with stage IV disease were excluded from the study; therefore, the final number of eligible patients was 2200. The Table provides the summary characteristics of the patients included in this analysis. All patients were women and the mean (SD) age was 60.4 (11.9) years. Median (IQR) BMI was 27.1 (23.8-32.0), 1603 patients (72.9%) were postmenopausal, 1797 patients (81.8%) were parous, and 1242 patients (56.7%) were never smokers and 842 patients (38.4%) were former smokers. The sample included 291 Asian patients (13.2%), 166 Black patients (7.5%), 253 Hispanic patients (11.5%), 1439 White patients (65.4%), and 51 patients (2.3%) who identified as other race or ethnicity. In this population, 1295 patients (57.2%) had ERBB2-low tumors, and 1956 patients (88.9%) had hormone receptor–positive tumors. There were 1144 patients (52.0%) with hormone receptor–positive and ERBB2-low tumors, 812 patients (36.9%) with hormone receptor–positive and ERBB2-negative tumors, 115 patients (5.2%) with hormone receptor–negative and ERBB2-low tumors, and 129 patients (5.9%) with hormone receptor–negative and ERBB2-negative tumors. ERBB2-low was observed more often in patients with hormone receptor–positive tumors than in patients with hormone receptor–negative tumors (1144 of 1956 patients [58.4%] vs 115 of 244 patients [47.1%]; *P* < .001).

**Table.** Pathways Study Characteristics Stratified by ERBB2 and Hormone Receptor Status

Characteristic^a^	Overall			Hormone receptor–positive^b^			Hormone receptor–negative		
	Patients by ERBB2 status, No. (%)		*P* value^c^	Patients by ERBB2 status, No. (%)		*P* value^c^	Patients by ERBB2 status, No. (%)		*P* value^c^
	Low	Negative		Low	Negative		Low	Negative	
Total	1259 (57.2)	941 (42.8)	NA	1144 (58.4)	812 (41.6)	NA	115 (47.1)	129 (52.9)	NA
Age at diagnosis, mean (SD), y	60.4 (12.1)	60.4 (11.7)	.96	60.5 (12.3)	60.6 (11.7)	.85	59.7 (10.2)	59.1 (11.3)	.68
BMI, mean (SD)	28.6 (6.8)	28.6 (6.9)	.79	28.5 (6.7)	28.6 (6.6)	.74	29.5 (7.7)	29.1 (8.1)	.69
Menopause status									
Premenopausal	334 (26.5)	263 (27.9)	.46	310 (27.1)	229 (28.2)	.59	24 (20.9)	34 (26.4)	.31
Postmenopausal	925 (73.5)	678 (72.1)		834 (72.9)	583 (71.8)		91 (79.1)	95 (73.6)	
Self-identified race and ethnicity									
Asian	181 (14.4)	110 (11.7)	.17	163 (14.2)	103 (12.7)	.28	18 (15.7)	7 (5.4)	.06
Black	102 (8.1)	64 (6.8)		78 (6.8)	41 (5.0)		24 (20.9)	23 (17.8)	
Hispanic	138 (11.0)	115 (12.2)		128 (11.2)	97 (11.9)		10 (8.7)	18 (14.0)	
White	806 (64.0)	633 (67.3)		747 (65.3)	556 (68.5)		59 (51.3)	77 (59.7)	
Other^d^	32 (2.5)	19 (2.0)		28 (2.4)	15 (1.8)		4 (3.5)	4 (3.1)	
Family history of BC									
No	1017 (81.4)	724 (77.2)	.02	923 (81.4)	620 (76.5)	.009	94 (81.7)	104 (81.2)	.92
Yes	232 (18.6)	214 (22.8)		211 (18.6)	190 (23.5)		21 (18.3)	24 (18.8)	
Parity, mean (SD)	1.9 (1.4)	2.1 (1.5)	.09	1.9 (1.5)	2.0 (1.4)	.08	2.1 (1.3)	2.1 (1.6)	.92
Smoking status									
Never	706 (56.4)	536 (57.0)	.55	642 (56.5)	460 (56.7)	.81	64 (55.7)	76 (58.9)	.53
Former	489 (39.1)	353 (37.6)		444 (39.1)	310 (38.2)		45 (39.1)	43 (33.3)	
Current	57 (4.6)	51 (5.4)		51 (4.5)	41 (5.1)		6 (5.2)	10 (7.8)	
Breastfeeding									
No	504 (40.0)	372 (39.5)	.81	452 (39.5)	317 (39.0)	.83	52 (45.2)	55 (42.6)	.68
Yes	755 (60.0)	569 (60.5)		692 (60.5)	495 (61.0)		63 (54.8)	74 (57.4)	
Histology									
IC-NST	841 (66.8)	638 (67.8)	.62	754 (65.9)	543 (66.9)	.66	87 (75.7)	95 (73.6)	.72
Other^e^	418 (33.2)	303 (32.2)		390 (34.1)	269 (33.1)		28 (24.3)	34 (26.4)	
Nottingham Grade									
1	250 (22.5)	185 (22.1)	.33	248 (24.5)	182 (25.0)	.86	2 (2.1)	3 (2.8)	.34
2	652 (58.7)	472 (56.4)		629 (62.0)	455 (62.4)		23 (23.7)	17 (15.7)	
3	209 (18.8)	180 (21.5)		137 (13.5)	92 (12.6)		72 (74.2)	88 (81.5)	
DCIS, mean (SD), %	10.2 (18.9)	9.5 (18.2)	.39	10.3 (18.9)	9.9 (18.7)	.66	9.8 (19.1)	6.7 (14.2)	.22
TILs									
Mean (SD)	12.1 (16.4)	12.8 (17.4)	.41	10.1 (13.8)	10.0 (13.9)	.81	32.8 (25.0)	31.4 (25.6)	.69
Low (≤30%)	1011 (91.2)	755 (90.2)	.47	10.1 (13.8)	10.0 (13.9)	.93	32.8 (25.0)	31.4 (25.6)	.6
High (>30%)	98 (8.8)	82 (9.8)		58 (5.7)	41 (5.6)		40 (41.2)	41 (37.6)	
Tumor stage									
I	693 (55.0)	544 (57.8)	.43	647 (56.6)	479 (59.0)	.55	46 (40.0)	65 (50.4)	.27
II	449 (35.7)	313 (33.3)		395 (34.5)	263 (32.4)		54 (47.0)	50 (38.8)	
III	117 (9.3)	84 (8.9)		102 (8.9)	70 (8.6)		15 (13.0)	14 (10.9)	
Adjuvant RT									
No	672 (53.4)	492 (52.3)	.61	592 (51.7)	409 (50.4)	.55	80 (69.6)	83 (64.3)	.39
Yes	587 (46.6)	449 (47.7)		552 (48.3)	403 (49.6)		35 (30.4)	46 (35.7)	
Adjuvant CT									
No	718 (57.3)	557 (59.3)	.35	695 (61.0)	527 (64.9)	.08	23 (20.2)	30 (23.4)	.54
Yes	536 (42.7)	383 (40.7)		445 (39.0)	285 (35.1)		91 (79.8)	98 (76.6)	
Hormone therapy									
No	238 (19.0)	214 (23.0)	.02	124 (10.9)	89 (11.1)	.91	114 (99.1)	125 (99.2)	>.99
Yes	1016 (81.0)	717 (77.0)		1015 (89.1)	716 (88.9)		1 (0.9)	1 (0.8)	
Surgery									
No	17 (1.4)	23 (2.4)	.06	15 (1.3)	19 (2.3)	.09	2 (1.7)	4 (3.1)	.69
Yes	1242 (98.6)	918 (97.6)		1129 (98.7)	793 (97.7)		113 (98.3)	125 (96.9)	

Abbreviations: BC, breast cancer; BMI, body mass index (calculated as weight in kilograms divided by height in meters squared); CT, chemotherapy; DCIS, ductal carcinoma in situ; ERBB2, human epidermal growth factor receptor 2; IC-NST, invasive carcinoma of no special type; NA, not applicable; RT, radiation therapy; TILS, tumor-infiltrating lymphocytes.

^a^ Missingness of covariates as follows: BMI, 1 patient; family history, 10 patients; parity, 3 patients; smoking status, 7 patients; Nottingham Grade, 148 patients; TILs, 150 patients; adjuvant CT, 5 patients; hormone therapy, 5 patients.

^b^ Defined as the presence of estrogen or progesterone receptors.

^c^ Assessed using Welch 2-sample *t* test for continuous variables and Pearson χ^2^ and Fisher exact test for categorical variables.

^d^ Includes American Indian or Alaskan Native and Pacific Islander.

^e^ Includes invasive lobular carcinoma, mixed IC-NST and invasive lobular carcinoma, mixed IC-NST and low risk, mixed IC-NST and high risk, mixed mucinous micropapillary, micropapillary, mucinous, metaplastic, tubular, cribriform, apocrine, and other special type.

Among the variables examined, only family history of BC was significantly associated with ERBB2-low status. Patients with ERBB2-low tumors were less likely than those with ERBB2-negative BC to have family history of BC (232 of 1259 patients [18.6%] vs 214 of 941 patients [22.8%]; *P* = .02). When patients were stratified by hormone receptor status, family history maintained its significance in the hormone receptor–positive group (211 of 1144 patients [18.6%] vs 190 of 812 patients [23.5%]; *P* = .009) but not in the hormone receptor–negative group (21 of 115 patients [18.3%] vs 24 of 129 patients [18.8%]; *P* = .92). No statistically significant differences were observed in other variables examined according to ERBB2-low vs ERBB2-negative status, including menopausal status, parity, smoking history, breast feeding, or BMI (Table).

ERBB2-low was detected in 18 of 25 Asian patients (72.0%), 24 of 47 Black patients (51.1%), 10 of 28 Hispanic patients (35.7%), 59 of 136 White patients (43.4%), and 4 of 8 patients (50.0%) with other reported race or ethnicity (*P* = .06). Furthermore, when Asian patients were compared with the rest of the SIRE groups, they had higher incidence of ERBB2-low (18 of 25 patients [72.0%] vs 92 of 211 patients [43.6%]; *P* = .01). (Table; eFigure 1 in Supplement 1).

### Correlations of ERBB2 Status With the Histologic Features

When the tumors were classified into IC-NST vs others, no statistically significant differences were observed by ERBB2 expression (Table). However, when each special type was separately analyzed, ERBB2-low was less often found in mucin-producing carcinomas (pure mucinous or mixed with micropapillary) vs nonmucinous tumors (15 of 39 patients [38.5%] vs 1244 of 2161 patients [57.6%]; *P* = .02). Metaplastic carcinoma tended to be ERBB2-negative, as only 1 of 8 patients had ERBB2-low tumors (eTable 1 in Supplement 1). Other histologic features, such as Nottingham grade and the percentage of DCIS, had no statistically significant associations with ERBB2 (Table).

No statistically significant associations were found between higher TILs and younger age, premenopausal status, Black SIRE, Hispanic SIRE, higher BMI, negative hormone receptor status, IC-NST, higher Nottingham grade, or more advanced tumor stage (eTable 2 in Supplement 1). Further, no statistically significant difference in TILs was noted between ERBB2-negative and ERBB2-low in hormone receptor–positive or hormone receptor–negative groups (eTable 3 and eFigure 2 in Supplement 1).

### Associations of ERBB2-Low Status With BC Survival Outcomes

The median (range) of follow-up was 10.4 (8.8 to 13.1) years. During the follow-up period, 473 deaths (21.5% of the cohort) occurred, including 198 deaths (9.0%) due to BC. Local or distant recurrence developed in 295 patients (13.4%).

**Figure 1.**
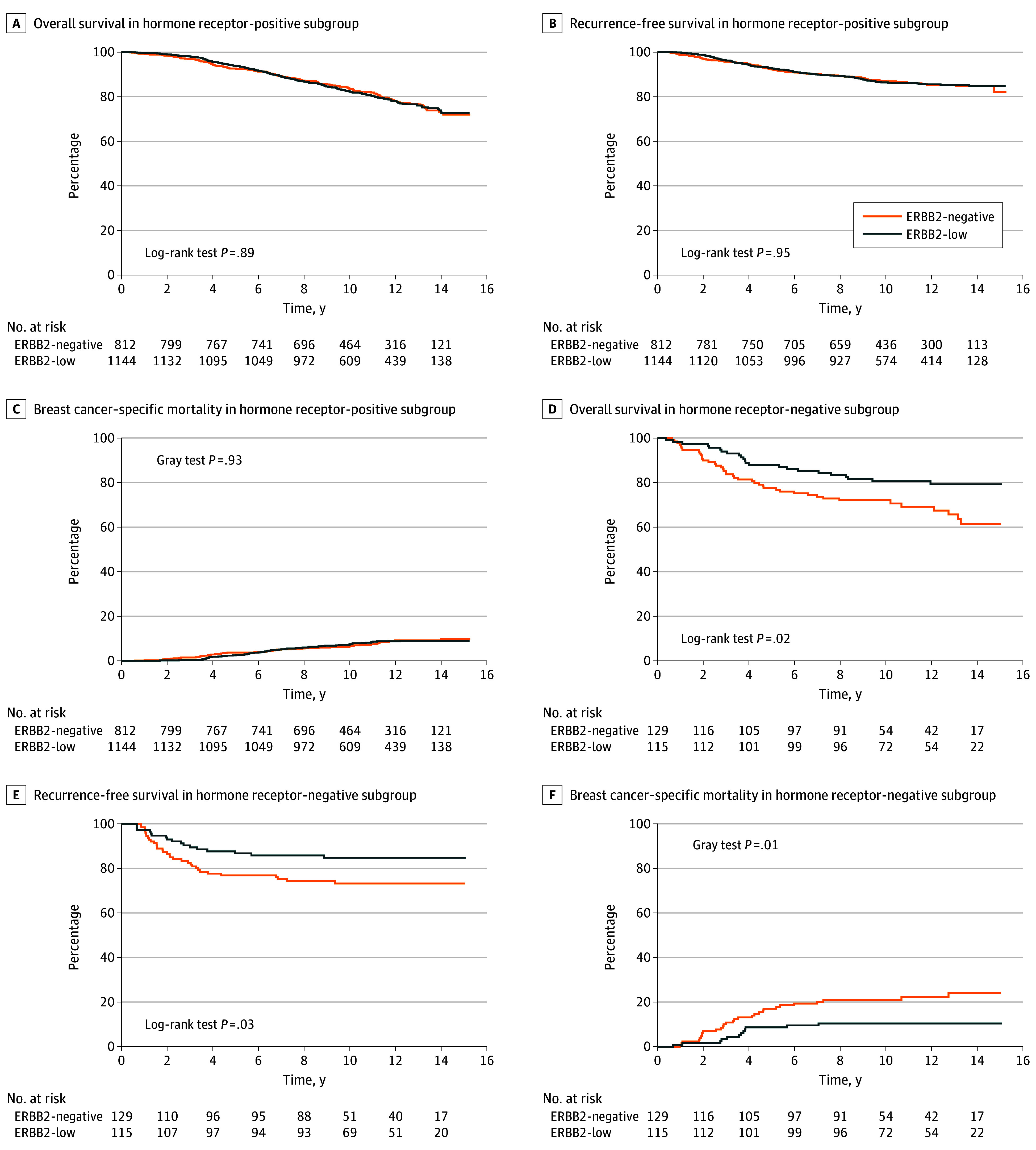
Kaplan-Meier and Cumulative Incidence Curves by Hormone Receptor Subgroups ERBB2 indicates human epidermal growth factor receptor 2.

Univariable analyses revealed patients with ERBB2-low tumors had no statistically significant difference in survivals when all patients were included (eFigure 3 in Supplement 1), or within the hormone receptor–positive subgroup (Figures 1A-C). Patients with ERBB2-low tumors had significantly better survival compared with those with ERBB2-negative tumors, in the hormone receptor–negative subgroup (Figures 1D-F; eTable 4 and eTable 5 in Supplement 1).

**Figure 2.**
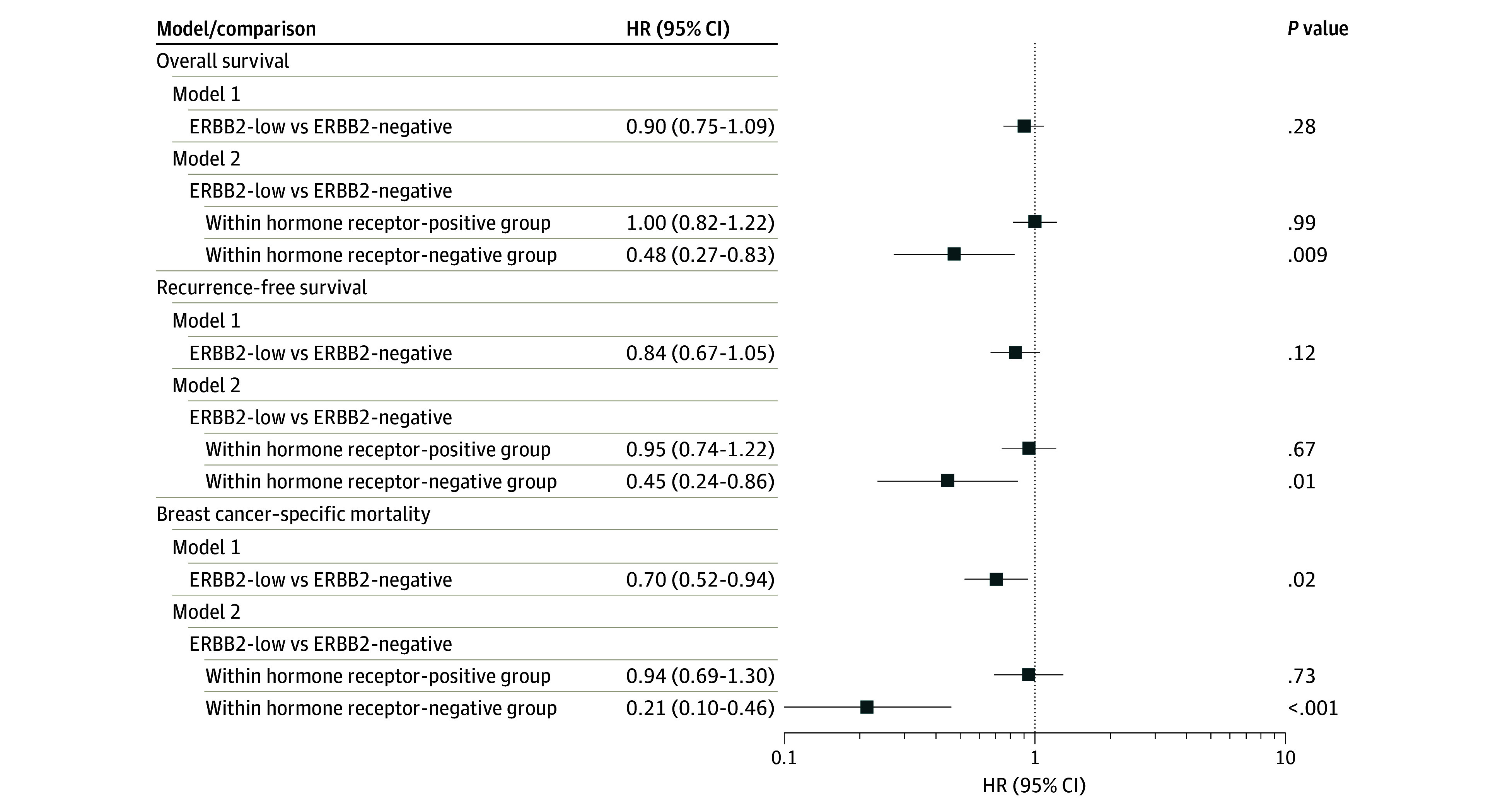
Associations of Human Epidermal Growth Factor Receptor 2 (ERBB2)-Low vs ERBB2-Negative in Overall Survival, Recurrence-Free Survival, and Breast Cancer–Specific Mortality With Non–Breast Cancer–Specific Mortality as a Competing Event Model 1 was stratified by hormone receptor status and adjusted for age at diagnosis, stage, radiation, chemotherapy, hormone therapy, and surgery. Model 2 additionally included an interaction between ERBB2 and hormone receptor status allowing for different coefficients for ERBB2 within hormone receptor strata. HR indicates hazard ratio.

In multivariable analyses, the ERBB2-low group had better BCSM compared with the ERBB2-negative group (subdistribution hazard ratio [HR], 0.70; 95% CI, 0.52-0.94; *P* = .02) in model 1. When considering the association of ERBB2 within hormone receptor strata in model 2, ERBB2-low status was not associated with a difference in survival within patients with hormone receptor–positive tumors. Within the hormone receptor–negative group, ERBB2-low status was associated with significantly better OS (HR, 0.48; 95% CI, 0.27-0.83; *P* = .009), RFS (HR, 0.45; 95% CI, 0.24-0.86; *P* = .02), and BCSM (subdistribution HR, 0.21; 95% CI, 0.10-0.46 *P* < .001) (Figure 2). Analyses within SIRE groups showed improved RFS for Black patients when comparing those with ERBB2-low tumors with those with ERBB2-negative tumors in model 1 (HR, 0.44; 95% CI, 0.21-0.90; *P* = .03) and model 2 (HR, 0.44; 95% CI, 0.21-0.94; *P* = .04) (eTable 6 in Supplement 1).

### Associations of TILs Within ERBB2-Low and ERBB2-Negative Tumors

The clinical significance of TILs was examined within ERBB2 and hormone receptor subgroups. We found most patients (74.2%) with hormone receptor–positive tumors had 10% TILs or less (eTable 2 in Supplement 1). Within the ERBB2-low and hormone receptor–negative subgroup, a 10% increase in TILs was associated with 35% reduced risk of RFS (HR, 0.65; 95% CI, 0.46-0.92; *P* = .02) and the 23% reduced risk of BCSM (subdistribution HR, 0.77; 95% CI, 0.62-0.96; *P* = .02) in age adjusted models. Adjustment for relevant clinical characteristics in model 2 revealed a 10% increase in TILs was associated with 41% reduced risk of RFS (HR, 0.59; 95% CI, 0.40-0.88; *P* = .009) (eTable 7 in Supplement 1). There were no statistically significant associations with TILs in any of the other groups, including the hormone receptor–negative and ERBB2-negative subgroup.

**Figure 3.**
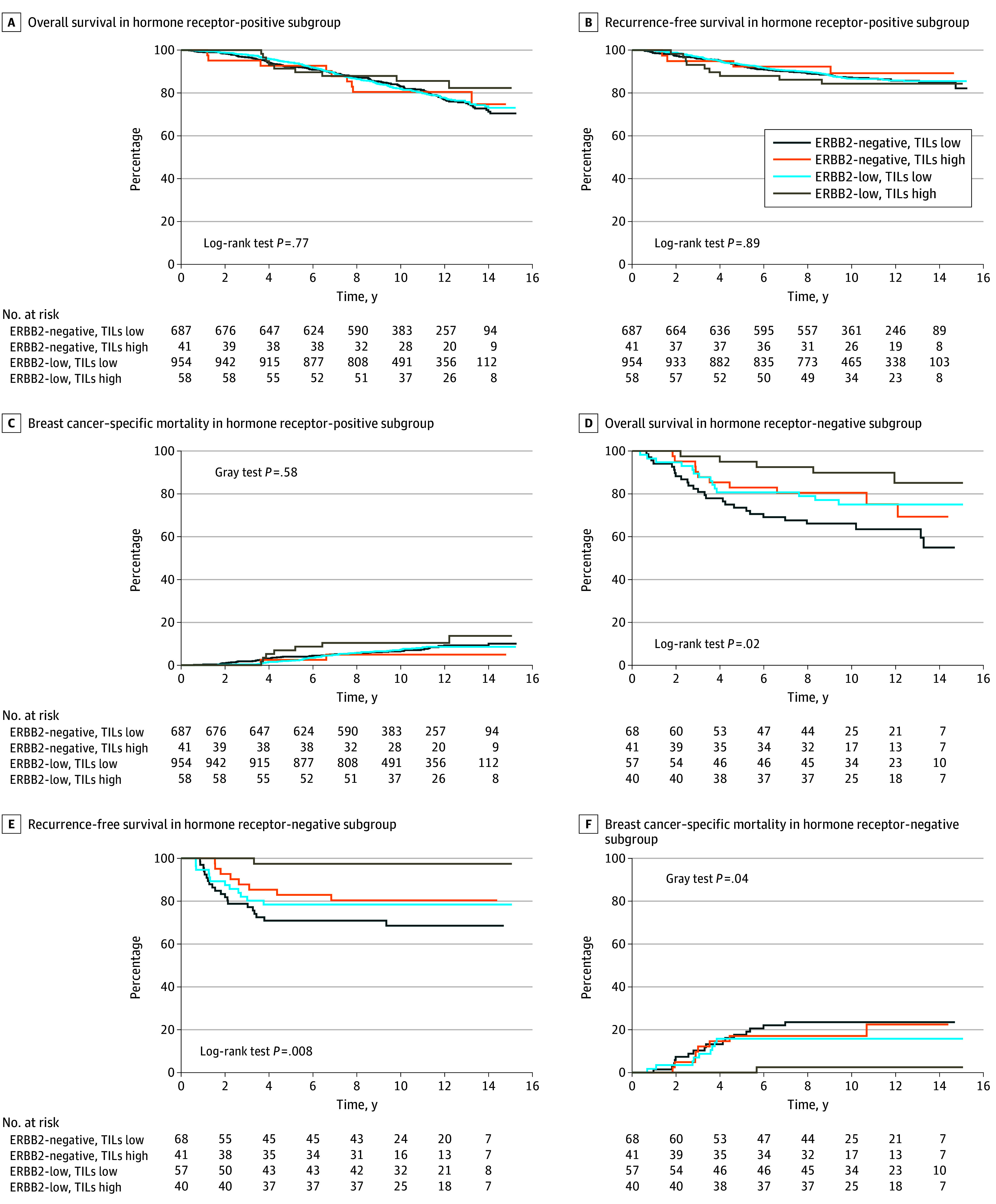
Kaplan-Meier and Cumulative Incidence Curves for Tumor Infiltrating Lymphocytes (TILs) and Human Epidermal Growth Factor Receptor 2 (ERBB2) Status by Hormone Receptor–Positive TIL status was defined as 30% or less, TILs low; more than 30%, TILs high.

**Figure 4.**
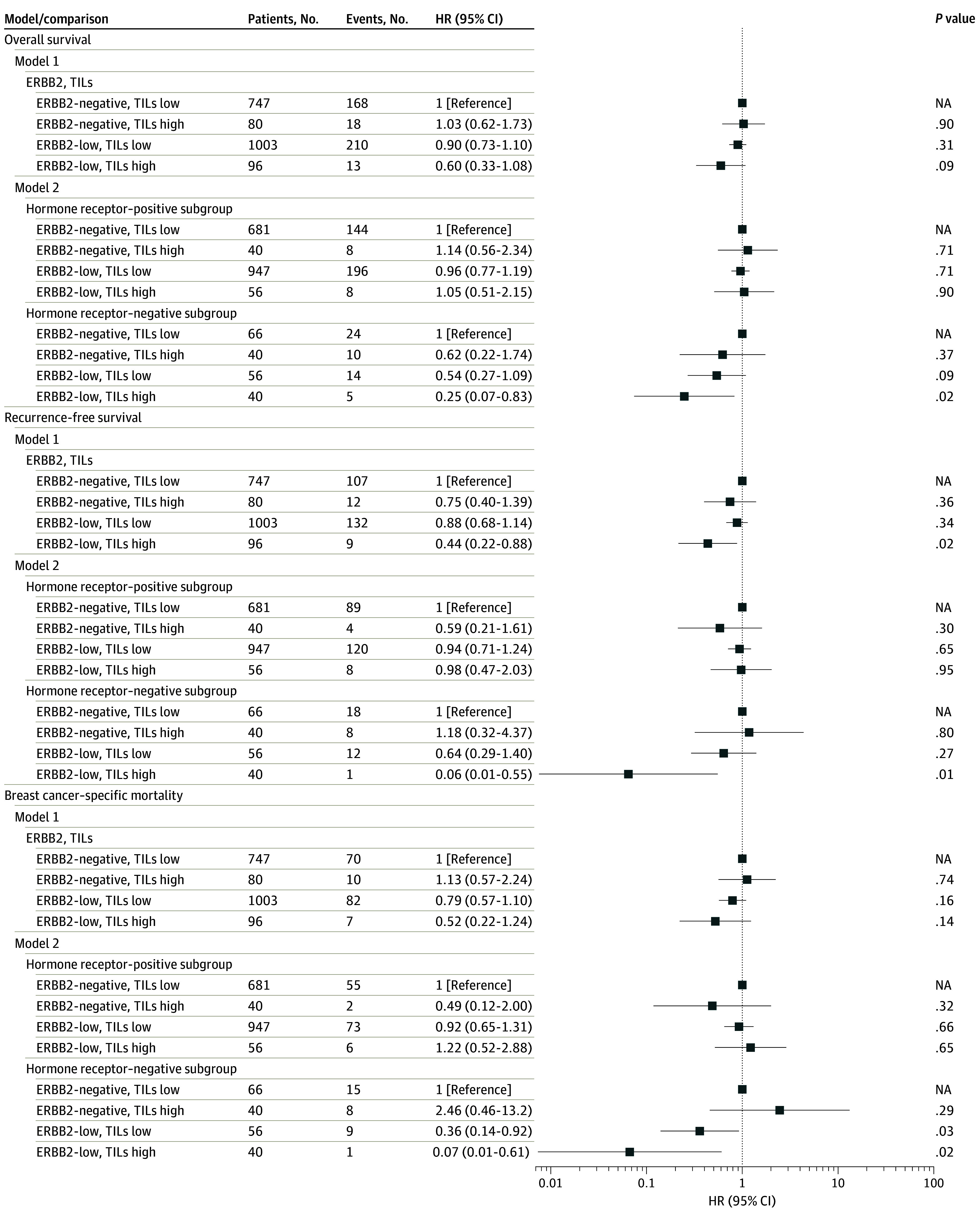
Associations of Human Epidermal Growth Factor Receptor 2 (ERBB2) and Tumor Infiltrating Lymphocytes (TILs) Status With Overall Survival, Recurrence-Free Survival, and Breast Cancer–Specific Mortality With Non–Breast Cancer–Specific Mortality as a Competing Event TIL status was defined as 30% or less, TILs low; more than 30%, TILs high. Model 1 stratified by hormone receptor status and adjusted for age at diagnosis, stage, radiation, chemotherapy, hormone therapy, and surgery. Model 2 additionally included an interaction among ERBB2, human epidermal growth factor receptor 2; ERBB2, TILs and hormone receptor status allowing for different coefficients for ERBB2 and TILs within hormone receptor strata. HR indicates hazard ratio; NA, not applicable.

Finally, a 30% cutoff was used to define low vs high TILs and was combined with ERBB2 using ERBB2-negative and low TILs as the referent group. ERBB2-low and high TILs was associated with better RFS in model 1 (HR, 0.44; 95% CI, 0.22-0.88; *P* = .02). In model 2, patients with ERBB2-low, high TILs, and hormone receptor–negative tumors had better survival across all 3 outcomes compared with patients with ERBB2-negative, low TILs, and hormone receptor–negative tumors; however, there was only 1 event within this group for RFS and BCSM. Additionally, the ERBB2-low and low TILs subgroup had improved BCSM (subdistribution HR, 0.36; 95% CI, 0.14-0.92; *P* = .03) (Figure 3 and Figure 4; eFigure 4 in Supplement 1.

## Discussion

To our knowledge, this cohort study is the first study to comprehensively evaluate the associations between ERBB2-low status and epidemiologic and histopathologic variables. There were differences in hormone receptor status, family history, and SIRE between ERBB2-low and ERBB2-negative status. Furthermore, within hormone receptor–negative tumors, ERBB2-low status was associated with better survival compared with ERBB2-negative status. These findings suggest that ERBB2-low might be a distinct biologic and clinical entity. These results have valuable implications for future classification of the disease, overall understanding of these subtypes, and the development of new therapeutic agents and targeted therapies, particularly for ERBB2-negative tumors.

The survival significance of ERBB2-low has been subject to substantial research with inconsistent results. Some studies found no survival differences comparing patients with ERBB2-low tumors with patients with ERBB2-negative tumors,^14,15,16,17,18,19,20,21,22^ while others found ERBB2-low to be significant in RFS, BCSM, and OS.^23,24,25,26,27,28,29^ However, in several meta-analyses, significant differences were present in clinical outcomes between ERBB2-low and ERBB2-negative tumors.^30,31,32,33,34,35^ We found significant OS benefit associated with ERBB2-low compared with ERBB2-negative in the hormone receptor–negative BC group (ie, TNBC). We also evaluated the significance of TILs in all subgroups and found that for every 10% increase in TILs, there was a significant increase in the RFS and BCSM in the hormone receptor–negative and ERBB2-low group but not in any other groups, including the hormone receptor–negative and ERBB2-negative group.

To our knowledge, only 1 study to date found TILs to be associated with survival in the ERBB2-low setting. Sun et al^36^ found patients with ERBB2-low BC, but not patients with ERBB2-negative BC, with TILs more than 10% had better RFS. Then they analyzed the subgroups and found that patients with hormone receptor–positive, ERBB2-low BC and patients with hormone receptor–negative and ERBB2-negative BC with TILs more than 10% had better RFS. They did not perform an analysis on hormone receptor–negative and ERBB2-low BC, a subgroup in which we found an association for TILs.^36^ It is known now after an extensive number of studies that TILs are associated with better survival in TNBC and to a lesser extent in ERBB2-positive BC, with fewer studies supporting the relevance of TILs in luminal (ie, hormone receptor–positive and ERBB2-negative) BC.^5,6^ The level of TILs in luminal-type BC is usually low. As most patients with hormone receptor-positive tumors had few TILs, this may explain the insignificance of TILs in the hormone receptor-positive group, unlike what Sun et al^36^ found.

As noted, the studies that evaluated ERBB2 status and TILs found that ERBB2-low and high TILs were associated with better clinical outcomes. We found similar results, but we went further and evaluated 4 subgroups. It was expected that the ERBB2-low and high TILs subgroup would have better outcomes than the ERBB2-negative and low TILs subgroup, although only a single event occurred in the former subgroup, precluding definitive conclusion. Interestingly, we found that the ERBB2-low and low TILs subgroup had better BCSM than the ERBB2-negative and low TILs subgroup.

The reasons for our observed differences in the BC outcomes based on ERBB2 expression (low vs negative) are unclear. Many investigators attribute it to other coexisting risk factors, as ERBB2-low BC is more likely to have low Ki-67,^24^ lower Nottingham grade, and more likely to be luminal type.^18^ However, this does not explain why there was a difference between ERBB2-low and ERBB2-negative in TNBC in this study. In this specific group of tumors, we found variation in clinical outcomes based on the degree of TILs. However, it is unclear whether there is a biologic interaction between TILs and the tumor cells that have varying degrees of ERBB2 protein, or they each play an independent role in the outcome through different mechanisms. More studies are needed to elucidate these theories.

Our study design gave us the advantage of evaluating associations with epidemiologic factors and with clinical outcomes of patients with different SIRE. We found that Black women with ERBB2-low had better RFS than those who had ERBB2 negative tumors. When Asian women were compared with the other SIRE groups combined, a higher proportion of Asian patients had ERBB2-low tumors. Only a few studies that evaluated ERBB2-low in different SIRE groups analyzed survival outcomes in these groups. Peiffer et al^29^ evaluated ERBB2-low in a large retrospective group of patients using the National Cancer Database. They found that incidence of ERBB2-low was lower in Black and Hispanic patients than in non-Hispanic White patients, although in non-Hispanic Black patients, differences in rates of TNBC and other confounders were likely the source. The incidence of ERBB2-low in Asian patients, on the other hand, was approximately 50%, with no difference from the other SIRE groups.^29^ This is not consistent with our results, which suggest that Asian patients were more likely have ERBB2-low tumors compared with the other SIRE groups. Furthermore, reviewing studies conducted in Asia, the prevalence rate of ERBB2-low was consistently greater than 54% of the study sample. In 4 of 6 studies, the rates were between 61.7% and 73.7%.^14,16,22,23,27,28^ While not evaluated as such clinically, the presence of the *ERBB2* gene and ERBB2 protein is a continuous variable in which the *ERBB2* gene copy number could reach up to 25 to 50 per cell, and the protein receptor could increase up to 40 to 100-fold, resulting in up to 2 million receptors.^37^ ERBB2-positive (3+ by IHC or FISH^+^) tumors are encountered more often in Asian patients than in other SIRE groups.^38^ We thus infer that Asian patients with BC are more likely to have ERBB2-low tumors than patients with BC in other SIRE groups, and this may be associated with differences in survival outcomes.

An association between family history of BC and ERBB2-low status was also observed in our study. Women with hormone receptor–positive BC, but not those with hormone receptor–negative BC, and women with ERBB2-low tumors were less likely than those with ERBB2-negative BC to have family history of BC. To our knowledge, only 1 study has performed a similar analysis and also found the same results.^14^ It has been described that family history does not play a role in ERBB2-positive BC.^39^ No clear explanation exists as to why family history is associated with ERBB2-low within the hormone receptor–positive group but not the hormone receptor–negative group. More studies are encouraged.

With regard to histologic subtypes and their association with ERBB2-low status, we found that tumors that produce mucin (mucinous or mixed mucinous micropapillary) had a lower rate of ERBB2-low expression than the other tumor types combined. Only 8 metaplastic carcinoma tumors were identified, most of which were ERBB2-negative. This information could be useful in patient treatment when anti–ERBB2-low therapy is considered and the patient has metastatic carcinoma with the primary tumor being mucin-producing or metaplastic BC. However, the number of patients in each of these 2 categories was very small. Only a single study by Peiffer et al^29^ evaluated the histologic subtype correlation with ERBB2-low. However, the study by Peiffer et al^29^ was retrospective, and the tumor classification was either not properly reported, or the reporting pathologist followed certain recommendations at the time of tumor evaluation. Nonetheless, Peiffer et al^29^ found that ERBB2-low was less common in certain subtypes compared with IC-NST including mucinous, lobular, mixed ductal and lobular, and metaplastic subtypes.

### Limitations and Strengths

This study has several limitations. ERBB2 status was abstracted from the pathology report. While this process ensured data consistency across the entire cohort diagnosed from 2005 to 2013, many patients were excluded because ERBB2-negative status (IHC score of 0+ or 1+) was not properly defined. In addition, hormone receptor–low data were not available, yet given its generally low incidence, this absence was unlikely to have significantly affected our results. Further, the number of patients with TNBC was relatively small. Although an experienced breast pathologist evaluated TILs following the published guidelines, the limitation of manual scoring should be acknowledged. Additionally, since we observed significant differences in clinical outcomes for ERBB2 status in TNBC, future work should examine the association between molecular subtypes and both TILs and ERBB2 status.

Despite these limitations, this study has several strengths compared with other studies examining ERBB2-low and clinical outcomes. As a prospective cohort study, patients were identified by daily searches of Systematized Nomenclature of Medicine codes, with enrollment into the study soon after diagnosis. After informed consent, an extensive baseline interview was conducted with the patients, enabling investigation of the association of various factors with ERBB2-low status. As all study participants had health insurance and received their health care, including cancer care, through KPNC, access to health care and its potential influence on outcomes is minimized. While an insured population may differ from a noninsured population, KPNC provides insurance and health care to more than 30% of the population in its 20-county service area, and thus its membership is largely representative of the general population, enhancing generalizability of findings related to biological characteristics of BC tumors. Additionally, pathology slides were centrally reviewed by an experienced breast pathologist following current recommendations, including histologic classification by current World Health Organization guidelines, ensuring changes in pathology standards over time did not affect classification of tumor histologic subtype, ERBB2 status, or measurement of TILs.^9,10^

## Conclusions

This cohort study found that BC with ERBB2-low was different from ERBB2-negative in a few clinical, pathological, and epidemiological aspects, suggesting ERBB2-low BC might be a unique entity. These results may play a role in future classification of the disease and the development of new therapeutic targeted therapies, particularly for ERBB2-negative tumors. Further studies with similar design are needed to validate our findings.

## Notes

Supplement 1. **eFigure 1**. Self-Identified Race and Ethnicity and ERBB2 Status **eFigure 2**. Histogram Showing TILs Distribution **eFigure 3**. Kaplan-Meier and Cumulative Incidence Curves for Overall Group **eFigure 4**. Kaplan-Meier and Cumulative Incidence Curves for TILs and ERBB2 for Overall Group **eTable 1.** Histologic Subtypes by ERBB2 Status **eTable 2.** Pathways Characteristics by TILs Low vs High **eTable 3.** TILs Distribution Within ERBB2 and HR **eTable 4.** Univariate Associations of Clinical and Pathological Variables and Survival Within HR+ Tumors **eTable 5.** Univariate Associations of Clinical and Pathological Variables and Survival Within HR- Tumors **eTable 6.** Associations of ERBB2 Status and Breast Cancer Outcomes Within Self-Identified Racial and Ethnic Groups **eTable 7.** Associations of a 10% Increase in TILs and Breast Cancer Outcomes Within ERBB2 and HR Subgroups

Supplement 2.
Data Sharing Statement
