# Supplementary material for: Clinical, Epidemiologic, and Pathologic Significance of ERBB2-Low Expression in Breast Cancer
Source: JAMA Netw Open. 2024 Mar 22;7(3):e243345. doi: 10.1001/jamanetworkopen.2024.3345 (PMC10960203; doi:10.1001/jamanetworkopen.2024.3345)
Supplement: Supplement 1. — eFigure 1. Self-Identified Race and Ethnicity and ERBB2 Status eFigure 2. Histogram Showing TILs Distribution eFigure 3. Kaplan-Meier and Cumulative Incidence Curves for Overall Group eFigure 4. Kaplan-Meier and Cumulative Incidence Curves for TILs and ERBB2 for Overall Group eTable 1. Histologic Subtypes by ERBB2 Status eTable 2. Pathways Characteristics by TILs Low vs High eTable 3. TILs Distribution Within ERBB2 and HR eTable 4. Univariate Associations of Clinical and Pathological Variables and Survival Within HR+ Tumors eTable 5. Univariate Associations of Clinical and Pathological Variables and Survival Within HR- Tumors eTable 6. Associations of ERBB2 Status and Breast Cancer Outcomes Within Self-Identified Racial and Ethnic Groups eTable 7. Associations of a 10% Increase in TILs and Breast Cancer Outcomes Within ERBB2 and HR Subgroups [file jamanetwopen-e243345-s001.pdf]

Supplemental Online Content

Khoury T, Mendicino L, Ondracek RP, et al. Clinical, epidemiologic, and pathologic significance of ERBB2-low expression in breast cancer. *JAMA Netw Open*. 2024;7(3):e243345. doi:10.1001/jamanetworkopen.2024.3345

eFigure 1. ERBB2 Status by Self-Identified Race and Ethnicity

eFigure 2. Tumor-Infiltrating Lymphocytes (TILs) Distribution, by Hormone Receptor and ERBB2 Status

eFigure 3. Kaplan-Meier Curves for Overall and Recurrence-Free Survival, and Cumulative Incidence Curves for Breast Cancer-Specific Mortality, by ERBB2 Status

eFigure 4. Kaplan-Meier Curves for Overall and Recurrence-Free Survival, and Cumulative Incidence Curves for Breast Cancer-Specific Mortality, by ERBB2 and TILs Status

eTable 1. Histologic Subtypes by ERBB2 Status

eTable 2. Pathways Study Participant Characteristics by TILs Category (Low or High)

eTable 3. TILs Distribution by ERBB2 and Hormone Receptor Status

eTable 4. Univariate Associations of Clinical and Pathological Variables and Survival Outcomes Within Hormone Receptor-Positive Tumors

eTable 5. Univariate Associations of Clinical and Pathological Variables and Survival Outcomes Within Hormone Receptor-Negative Tumors

eTable 6. Associations of ERBB2 Status and Breast Cancer Outcomes by Self-Identified Racial and Ethnic Groups

eTable 7. Associations of a 10% Increase in TILs and Breast Cancer Outcomes by ERBB2 and Hormone Receptor Subgroups

This supplemental material has been provided by the authors to give readers additional information about their work.

© 2024 [First author last name and initials or group name] et al. *[Journal name].*

eFigure 1. ERBB2 Status by Self-Identified Race and Ethnicity.

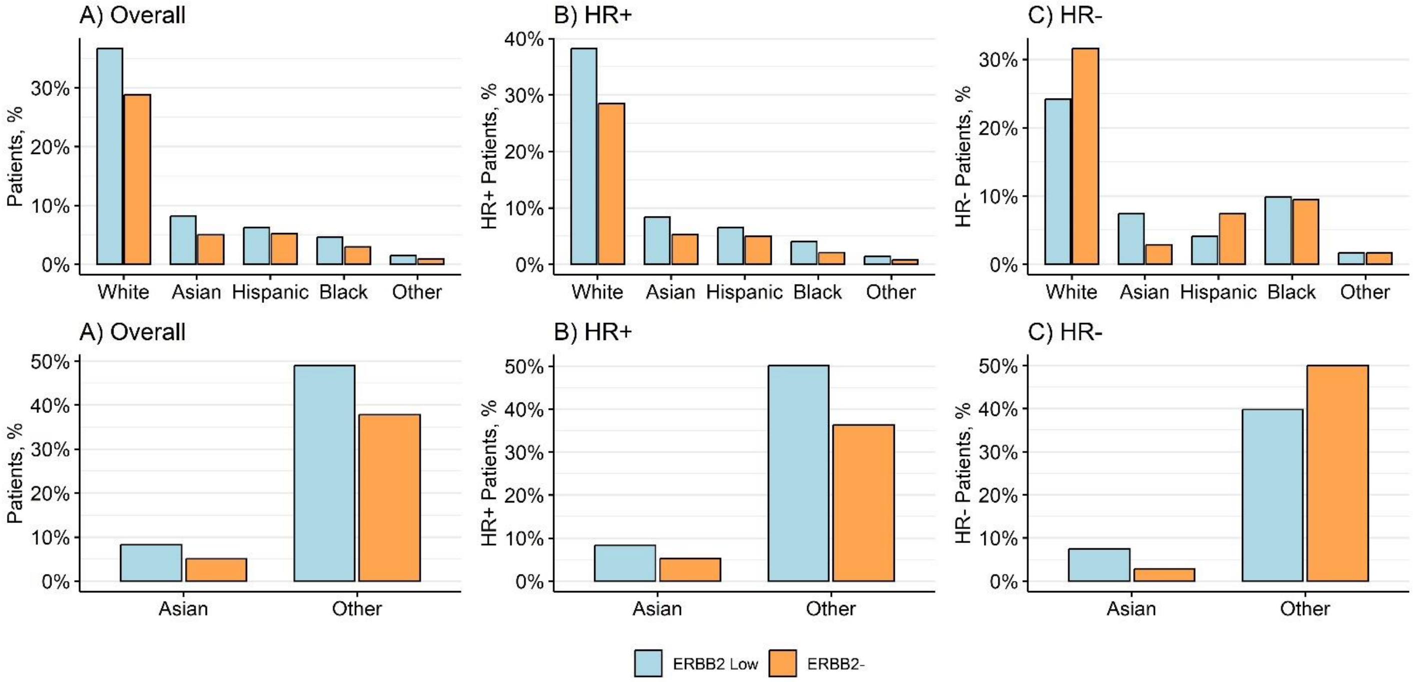

© 2024 [First author last name and initials or group name] et al. *[Journal name].*

eFigure 2. Tumor-Infiltrating Lymphocytes (TILs) Distribution, by Hormone Receptor and ERBB2 Status

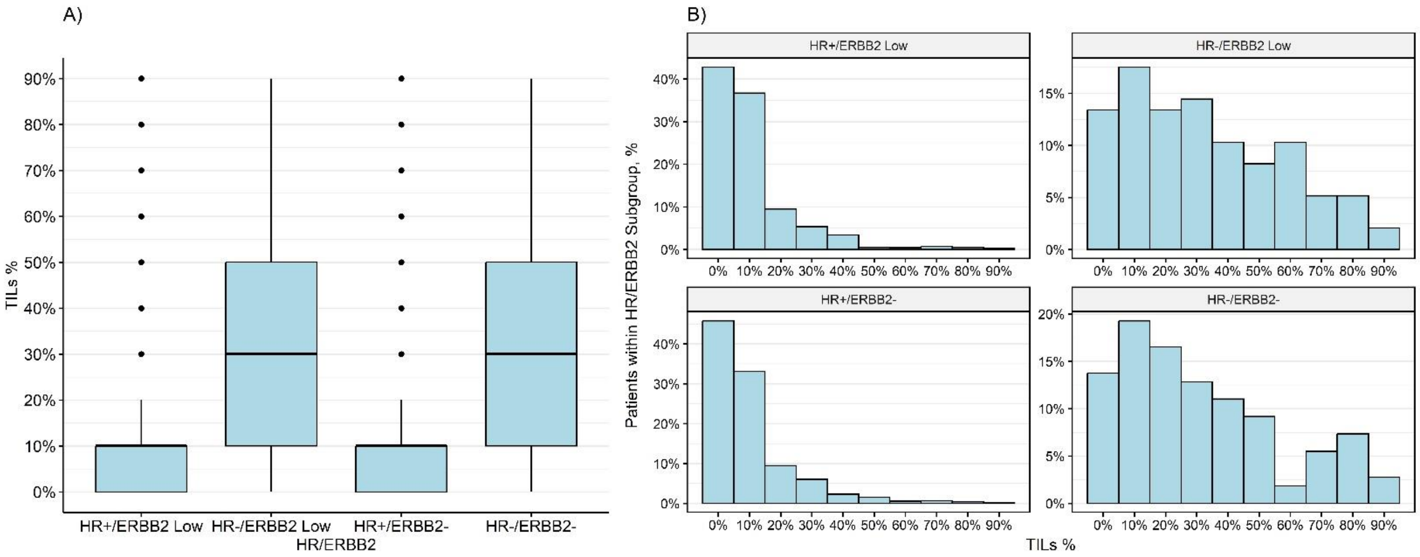

© 2024 [First author last name and initials or group name] et al. *[Journal name].*

eFigure 3. Kaplan-Meier Curves for Overall and Recurrence-Free Survival, and Cumulative Incidence Curves for Breast Cancer-Specific Survival, by ERBB2 Status.

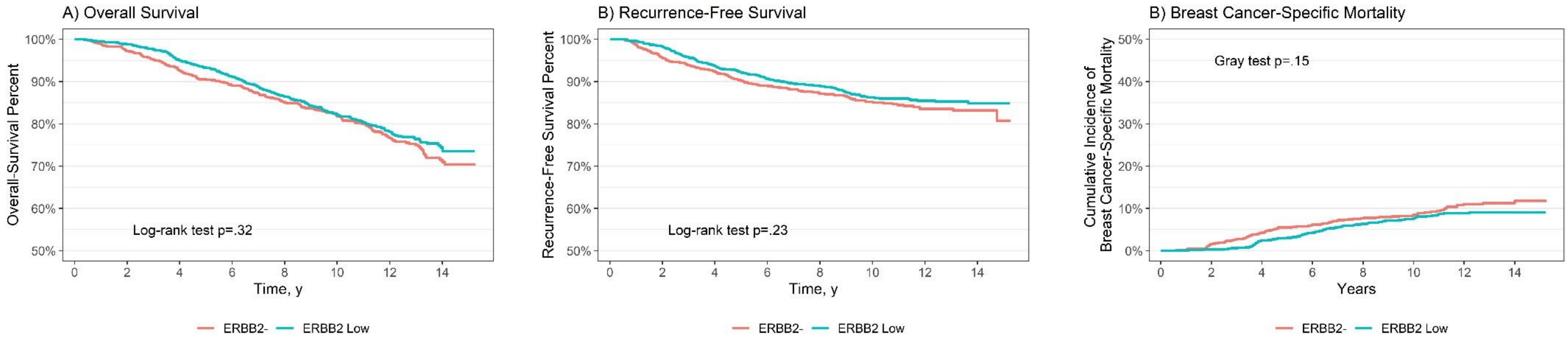

At Risk

ERBB2-	941	915	872	838	787	518	358	138
ERBB2 Low	1259	1244	1196	1148	1068	681	493	160

At Risk

ERBB2-	941	891	846	800	747	487	340	130
ERBB2 Low	1259	1227	1150	1090	1020	643	465	148

At Risk

ERBB2-	941	915	872	838	787	518	358	138
ERBB2 Low	1259	1244	1196	1148	1068	681	493	160

© 2024 [First author last name and initials or group name] et al. *[Journal name].*

eFigure 4. Kaplan-Meier Curves for Overall and Recurrence-Free Survival, and Cumulative Incidence Curves for Breast Cancer-Specific Survival, by ERBB2 and TILs Status

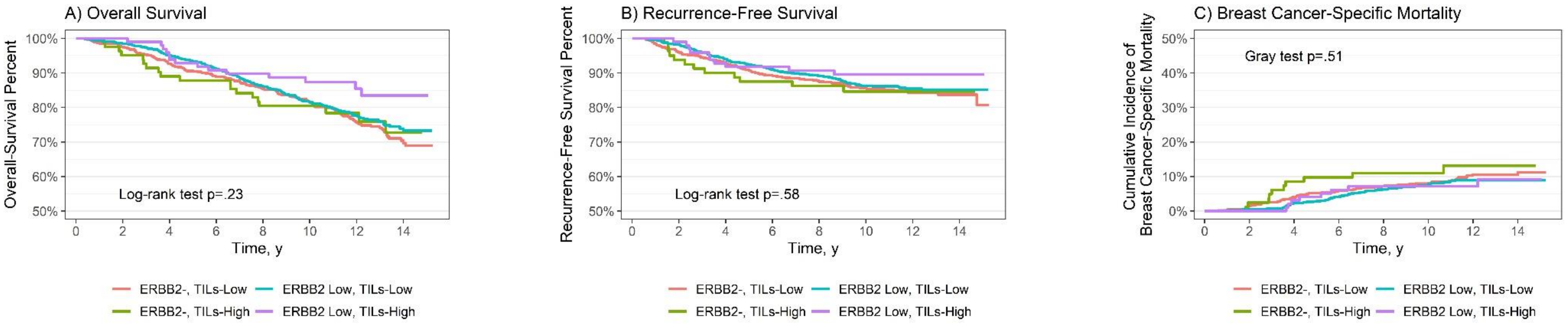

At Risk

ERBB2-, TILs-Low	755	736	700	671	634	408	278	101
ERBB2-, TILs-High	82	78	73	72	64	45	33	16
ERBB2 Low, TILs-Low	1011	996	961	923	853	525	379	122
ERBB2 Low, TILs-High	98	98	93	89	88	62	44	15

At Risk

ERBB2-, TILs-Low	755	719	681	640	600	385	266	96
ERBB2-, TILs-High	82	75	72	70	62	42	32	15
ERBB2 Low, TILs-Low	1011	983	925	878	815	497	359	111
ERBB2 Low, TILs-High	98	97	89	87	86	59	41	15

At Risk

ERBB2-, TILs-Low	755	736	700	671	634	408	278	101
ERBB2-, TILs-High	82	78	73	72	64	45	33	16
ERBB2 Low, TILs-Low	1011	996	961	923	853	525	379	122
ERBB2 Low, TILs-High	98	98	93	89	88	62	44	15

© 2024 [First author last name and initials or group name] et al. *[Journal name].*

eTable 1. Histologic Subtypes by ERBB2 Status

Characteristic	ERBB2-low	ERBB2-negative
Total	1,111 (57.0)*	837 (43.0)
IC-NST	841 (75.7)	638 (76.2)
ILC	111 (10.0)	96 (11.5)
Mixed IC-NST/ILC	15 (1.4)	7 (0.8)
Mixed IC-NST/low risk	40 (3.6)	26 (3.1)
Mixed IC-NST/high risk	21 (1.9)	6 (0.7)
Mixed mucinous micropapillary	4 (0.4)	8 (1.0)
Micropapillary	17 (1.5)	7 (0.8)
Mucinous	11 (1.0)	16 (1.9)
Metaplastic/	1 (0.1)	7 (0.8)
Tubular	24 (2.2)	17 (2.0)
Cribriform	10 (0.9)	3 (0.4)
Apocrine	7 (0.6)	1 (0.1)
Other-special type	9 (0.8)	5 (0.6)

*n (%), IC-NST, invasive carcinoma of no special type; ILC, invasive lobular carcinoma

© 2024 [First author last name and initials or group name] et al. *[Journal name].*

eTable 2. Pathways Study Participant Characteristics by TILs Category (Low or High)

Characteristic	Overall	Low (≤30%)	High (>30%)	p-value[2]
Total, n (%)	1,946	1,766 (90.1)	180 (9.9)	
Age at diagnosis (y), Mean (SD)	60.6 (11.8)	61.1 (11.7)	55.7 (12.0)	<.001
BMI, Mean (SD)	28.7 (6.9)	28.5 (6.8)	29.8 (8.0)	.04
Menopause status, n (%)				<.001
Pre-menopausal	515 (26.5)	442 (25.0)	73 (40.6)	
Post-menopausal	1,431 (73.5)	1,324 (75.0)	107 (59.4)	
SIRE, n (%)				<.001
White	1,281 (65.8)	1,198 (67.8)	83 (46.1)	
Asian	250 (12.8)	224 (12.7)	26 (14.4)	
Hispanic	222 (11.4)	188 (10.6)	34 (18.9)	
Black	146 (7.5)	116 (6.6)	30 (16.7)	
Other	47 (2.4)	40 (2.3)	7 (3.9)	
Family history, n (%)				.24
No	1,543 (79.8)	1,395 (79.4)	148 (83.1)	
Yes	391 (20.2)	361 (20.6)	30 (16.9)	
Parity, Mean (SD)	2.0 (1.4)	2.0 (1.4)	1.9 (1.4)	.51
Smoking Status, n (%)				0.08
Never	1,084 (55.9)	970 (55.1)	114 (63.7)	
Former	755 (38.9)	699 (39.7)	56 (31.3)	
Current	100 (5.2)	91 (5.2)	9 (5.0)	
Breastfeeding, n (%)				0.38
No	773 (39.7)	696 (39.4)	77 (42.8)	
Yes	1,173 (60.3)	1,070 (60.6)	103 (57.2)	
ER status, n (%)				<.001
Positive	1,739 (89.4)	1,641 (92.9)	98 (54.4)	
Negative	207 (10.6)	125 (7.1)	82 (45.6)	
PR status, n (%)				<.001
Positive	1,398 (71.8)	1,332 (75.4)	66 (36.7)	
Negative	548 (28.2)	434 (24.6)	114 (63.3)	
HR status, n (%)				<.001
HR-positive	1,740 (89.4)	1,641 (92.9)	99 (55.0)	
HR-negative	206 (10.6)	125 (7.1)	81 (45.0)	
ERBB2 status, n (%)				.47
ERBB2-low	1,109 (57.0)	1,011 (57.2)	98 (54.4)	
ERBB2-negative	837 (43.0)	755 (42.8)	82 (45.6)	
Histology, n (%)				<.001
IC-NST	1,476 (75.8)	1,309 (74.1)	167 (92.8)	
Other	470 (24.2)	457 (25.9)	13 (7.2)	
Nottingham Grade, n (%)				<.001
1	435 (22.4)	428 (24.2)	7 (3.9)	
2	1,120 (57.6)	1,082 (61.3)	38 (21.2)	
3	389 (20.0)	255 (14.4)	134 (74.9)	
DCIS %, Mean (SD)	9.9 (18.5)	10.0 (18.7)	8.3 (16.9)	.22
Stage, n (%)				<.001
I	1,095 (56.3)	1,021 (57.8)	74 (41.1)	
II	681 (35.0)	587 (33.2)	94 (52.2)	
III	170 (8.7)	158 (8.9)	12 (6.7)	
Radiation, n (%)				.005
No	1,017 (52.3)	905 (51.2)	112 (62.2)	
Yes	929 (47.7)	861 (48.8)	68 (37.8)	
Chemotherapy, n (%)				<.001
No	1,139 (58.7)	1,091 (61.9)	48 (27.0)	
Yes	801 (41.3)	671 (38.1)	130 (73.0)	
Hormone therapy, n (%)				<.001
No	383 (19.8)	290 (16.5)	93 (52.2)	
Yes	1,549 (80.2)	1,464 (83.5)	85 (47.8)	
Surgery, n (%)				>0.99
No	29 (1.5)	27 (1.5)	2 (1.1)	
Yes	1,917 (98.5)	1,739 (98.5)	178 (98.9)	

[1]Mean (SD); n (%)
[2]Welch Two Sample t-test; Pearson's Chi-squared test; Fisher's Exact Test

© 2024 [First author last name and initials or group name] et al. *[Journal name].*

eTable 3. TILs Distribution by ERBB2 and Hormone Receptor Status

Characteristic	Overall			Hormone Receptor-positive			Hormone Receptor-negative		
	ERBB2-low	ERBB2-negative	p-value[1]	ERBB2-low	ERBB2-negative	p-value[2]	ERBB2-low	ERBB2-negative	p-value[1]
Total	1,109 (57.0)	837 (43.0)		1,012 (58.2)	728 (41.8)		97 (47.1)	109 (52.9)	
TILs			.41			.81			.69
Mean (SD)	12.1 (16.4)	12.8 (17.4)		10.1 (13.8)	10.0 (13.9)		32.8 (25.0)	31.4 (25.6)	
Median (IQR)	10.0 (10.0)	10.0 (20.0)		10.0 (10.0)	10.0 (10.0)		30.0 (40.0)	30.0 (40.0)	
Range	0.0, 90.0	0.0, 90.0		0.0, 90.0	0.0, 90.0		0.0, 90.0	0.0, 90.0	
TILs, n (%)			.47			.93			.60
Low (<=30%)	1,011 (91.2)	755 (90.2)		954 (94.3)	687 (94.4)		57 (58.8)	68 (62.4)	
High (>30%)	98 (8.8)	82 (9.8)		58 (5.7)	41 (5.6)		40 (41.2)	41 (37.6)	

© 2024 [First author last name and initials or group name] et al. *[Journal name].*

eTable 4. Univariate Associations of Clinical and Pathological Variables and Survival Outcomes for Hormone Receptor-Positive Cases

Characteristic		Overall Survival		Recurrence-Free Survival		Breast Cancer-Specific Mortality	
	N	HR (95% CI)[1]	p-value	HR (95% CI)[1]	p-value	HR (95% CI)[1]	p-value
Age at Diagnosis (years)							
<50	394	—		—		—	
>50	1,562	2.68 (1.92 to 3.74)	<.001	0.94 (0.70 to 1.26)	.66	0.99 (0.67 to 1.46)	.96
BMI (kg/m^2)	1,955	1.02 (1.01 to 1.04)	.001	1.01 (0.99 to 1.03)	.28	1.03 (1.01 to 1.05)	.002
Menopause Status							
Pre-menopausal	539	—		—		—	
Post-menopausal	1,417	2.72 (2.04 to 3.62)	<.001	1.00 (0.76 to 1.31)	>.99	1.08 (0.76 to 1.54)	.67
Self-identified race/ethnicity							
White	1,303	—		—		—	
Asian	266	0.50 (0.34 to 0.72)	<.001	1.03 (0.72 to 1.48)	.85	1.08 (0.68 to 1.74)	.73
Hispanic	225	0.65 (0.46 to 0.93)	.02	0.88 (0.58 to 1.33)	.55	1.16 (0.71 to 1.89)	.56
Black	119	1.18 (0.81 to 1.72)	.38	1.42 (0.90 to 2.24)	.13	1.66 (0.95 to 2.91)	.07
Other	43	1.05 (0.56 to 1.97)	.88	1.29 (0.61 to 2.76)	.5	1.59 (0.65 to 3.87)	.31
Family History							
No	1,543	—		—		—	
Yes	401	1.15 (0.91 to 1.45)	.24	0.93 (0.68 to 1.26)	.62	0.94 (0.64 to 1.39)	.77
Parity	1,952	1.19 (1.13 to 1.27)	<.001	1.01 (0.93 to 1.10)	.79	1.03 (0.93 to 1.14)	.53
Smoking Status							
Never	1,102	—		—		—	
Former	754	1.38 (1.13 to 1.69)	.002	0.94 (0.73 to 1.21)	.62	0.85 (0.61 to 1.18)	.34
Current	92	1.79 (1.20 to 2.68)	.005	0.66 (0.32 to 1.34)	.25	0.74 (0.32 to 1.72)	.49
Breastfeeding							
No	769	—		—		—	
Yes	1,187	0.90 (0.74 to 1.09)	.28	1.02 (0.79 to 1.31)	.88	0.99 (0.72 to 1.35)	.93
ERBB2							
ERBB2-negative	812	—		—		—	
ERBB2-low	1,144	1.01 (0.83 to 1.23)	.89	0.99 (0.78 to 1.27)	.95	1.01 (0.74 to 1.39)	.94
Histology							
IC-NST	1,297	—		—		—	
Other	659	1.08 (0.89 to 1.33)	.44	1.19 (0.92 to 1.53)	.18	1.05 (0.75 to 1.45)	.79
Nottingham Grade							
1	430	—		—		—	
2	1,084	1.46 (1.12 to 1.92)	.006	2.60 (1.69 to 4.00)	<.001	3.98 (2.08 to 7.63)	<.001
3	229	1.73 (1.21 to 2.46)	.002	4.37 (2.69 to 7.11)	<.001	6.37 (3.12 to 13.0)	<.001
DCIS %	1,636	0.99 (0.99 to 1.00)	.04	1.00 (0.99 to 1.00)	.37	0.99 (0.98 to 1.01)	.35
TILs	1,740	0.93 (0.86 to 1.01)	.09	1.05 (0.96 to 1.14)	.32	1.05 (0.94 to 1.17)	.41
TILs							
Low (≤30%)	1,641	—		—		—	
High (>30%)	99	0.81 (0.51 to 1.31)	.4	1.02 (0.58 to 1.78)	.95	1.15 (0.58 to 2.26)	.7
Tumor Stage							
I	1,126	—		—		—	
II	658	1.36 (1.10 to 1.69)	.005	2.88 (2.17 to 3.84)	<.001	3.29 (2.22 to 4.87)	<.001
III	172	2.72 (2.06 to 3.59)	<0.001	6.32 (4.52 to 8.85)	<.001	9.55 (6.24 to 14.6)	<.001
Adjuvant RT							
No	1,001	—		—		—	
Yes	955	0.66 (0.54 to 0.81)	<.001	0.52 (0.40 to 0.67)	<.001	0.49 (0.35 to 0.68)	<.001
Adjuvant CT							
No	1,222	—		—		—	
Yes	730	0.84 (0.69 to 1.03)	.10	2.35 (1.83 to 3.01)	<.001	3.06 (2.21 to 4.25)	<.001
Hormone Therapy							
No	213	—		—		—	
Yes	1,731	0.54 (0.42 to 0.70)	<.001	0.57 (0.41 to 0.80)	<.001	0.83 (0.52 to 1.32)	.43
Surgery							
No	34	—		—		—	
Yes	1,922	0.37 (0.22 to 0.61)	<.001	0.26 (0.15 to 0.44)	<.001	0.21 (0.11 to 0.38)	<.001

[1]HR = Hazard Ratio, CI = Confidence Interval

© 2024 [First author last name and initials or group name] et al. *[Journal name].*

eTable 5. Univariate Associations of Clinical and Pathological Variables and Survival Outcomes within Hormone Receptor-Negative Cases

Characteristic	Overall Survival			Recurrence-Free Survival		Breast Cancer-Specific Mortality	
	N	HR (95% CI)[1]	p-value	HR (95% CI)[1]	p-value	HR (95% CI)[1]	p-value
Age at Diagnosis (years)							
<50	50	—		—		—	
>50	194	0.81 (0.45 to 1.44)	.47	0.67 (0.36 to 1.27)	.22	0.58 (0.30 to 1.14)	.12
BMI (kpg/m^2)	244	0.97 (0.94 to 1.01)	.15	0.99 (0.95 to 1.02)	.5	0.96 (0.92 to 1.01)	.12
Menopause Status							
Pre-menopausal	58	—		—		—	
Post-menopausal	186	0.70 (0.41 to 1.19)	.19	0.61 (0.33 to 1.10)	.1	0.50 (0.26 to 0.94)	.03
Self-identified race/ethnicity							
White	136	—		—		—	
Asian	25	0.22 (0.05 to 0.89)	.04	0.31 (0.07 to 1.29)	.11	0.41 (0.10 to 1.72)	.22
Hispanic	28	0.51 (0.20 to 1.30)	.16	1.06 (0.47 to 2.41)	.89	0.98 (0.37 to 2.60)	.97
Black	47	0.71 (0.36 to 1.37)	.3	0.81 (0.39 to 1.70)	.58	1.03 (0.49 to 2.18)	.93
Other	8	1.16 (0.36 to 3.74)	.8	0.53 (0.07 to 3.90)	.54	0.00 (0.00 to 0.00)	<.001
Family History							
No	198	—		—		—	
Yes	45	1.04 (0.55 to 1.94)	.91	0.88 (0.41 to 1.88)	.75	0.74 (0.31 to 1.76)	.5
Parity	244	1.05 (0.90 to 1.23)	.51	1.05 (0.88 to 1.25)	.59	1.04 (0.86 to 1.26)	.7
Smoking Status							
Never	140	—		—		—	
Former	88	1.14 (0.68 to 1.90)	.62	1.14 (0.63 to 2.04)	.67	0.92 (0.48 to 1.78)	.81
Current	16	0.90 (0.32 to 2.52)	.84	0.91 (0.28 to 2.99)	.87	1.05 (0.33 to 3.37)	.93
Breastfeeding							
No	107	—		—		—	
Yes	137	1.33 (0.81 to 2.20)	.26	1.38 (0.78 to 2.44)	.27	1.41 (0.75 to 2.66)	.28
ERBB2							
ERBB2-negative	129	—		—		—	
ERBB2-low	115	0.54 (0.33 to 0.91)	.02	0.53 (0.30 to 0.95)	.03	0.43 (0.22 to 0.84)	.01
Histology							
IC-NST	182	—		—		—	
Other	62	0.96 (0.54 to 1.68)	.88	1.12 (0.61 to 2.08)	.71	1.22 (0.63 to 2.37)	.56
Nottingham Grade							
3	160	—		—		—	
1	5	1.28 (0.31 to 5.28)	.73	0.00 (0.00 to Inf)	>.99	0.00 (0.00 to 0.00)	<.001
2	40	0.46 (0.20 to 1.08)	.08	0.40 (0.14 to 1.12)	.08	0.36 (0.11 to 1.15)	.08
DCIS %	184	0.98 (0.96 to 1.01)	.17	1.00 (0.99 to 1.02)	.63	0.98 (0.96 to 1.00)	.1
TILs	206	0.90 (0.81 to 1.01)	.07	0.84 (0.73 to 0.97)	.02	0.89 (0.79 to 1.00)	.05
TILs							
Low (≤30%)	125	—		—		—	
High (>30%)	81	0.52 (0.29 to 0.95)	.03	0.37 (0.18 to 0.78)	.009	0.53 (0.25 to 1.13)	.1
Tumor Stage							
I	111	—		—		—	
II	104	2.05 (1.15 to 3.66)	.02	2.33 (1.17 to 4.67)	.02	2.86 (1.27 to 6.43)	.01
III	29	4.39 (2.21 to 8.72)	<.001	6.05 (2.79 to 13.1)	<.001	8.60 (3.56 to 20.8)	<.001
Adjuvant RT							
No	163	—		—		—	
Yes	81	1.00 (0.59 to 1.67)	.99	1.25 (0.71 to 2.22)	.44	1.34 (0.72 to 2.49)	.36
Adjuvant CT							
No	53	—		—		—	
Yes	189	0.92 (0.52 to 1.63)	.77	1.44 (0.67 to 3.06)	.35	1.73 (0.73 to 4.07)	.21
Hormone Therapy							
No	239	—		—		—	
Yes	2	2.57 (0.36 to 18.6)	.35	3.18 (0.44 to 23.1)	.25	4.75 (0.41 to 55.6)	.21
Surgery							
No	6	—		—		—	
Yes	238	0.38 (0.12 to 1.20)	.1	0.27 (0.09 to 0.88)	.03	0.38 (0.07 to 1.93)	.24

[1]HR = Hazard Ratio, CI = Confidence Interval

© 2024 [First author last name and initials or group name] et al. *[Journal name].*

eTable 6. Associations of ERBB2 Status and Breast Cancer Survival Outcomes by Self-Identified Racial or Ethnic Groups

Model	Characteristic	Asian		Black		Hispanic		White	
		HR (95% CI)[1]	p-value	HR (95% CI)[1]	p-value	HR (95% CI)[1]	p-value	HR (95% CI)[1]	p-value
Overall Survival									
1	ERBB2-negative	—		—		—		—	
	ERBB2-low	0.96 (0.47 to 1.96)	.91	0.69 (0.37 to 1.28)	.23	0.75 (0.40 to 1.41)	.37	0.98 (0.79 to 1.21)	.85
2	ERBB2-negative	—		—		—		—	
	ERBB2-low	0.94 (0.45 to 1.96)	.87	0.73 (0.38 to 1.41)	.35	0.72 (0.37 to 1.40)	.34	1.01 (0.82 to 1.26)	.9
Recurrence-Free Survival									
1	ERBB2-negative	—		—		—		—	
	ERBB2-low	0.82 (0.43 to 1.56)	.54	0.44 (0.21 to 0.90)	.03	0.75 (0.38 to 1.49)	.41	0.98 (0.74 to 1.30)	.91
2	ERBB2-negative	—		—		—		—	
	ERBB2-low	0.81 (0.41 to 1.57)	.53	0.44 (0.21 to 0.94)	.04	0.77 (0.37 to 1.57)	.47	0.97 (0.73 to 1.29)	.83
Breast Cancer-Specific Mortality									
1	ERBB2-negative	—		—		—		—	
	ERBB2-low	1.13 (0.48 to 2.68)	.78	0.55 (0.24 to 1.25)	.15	0.67 (0.30 to 1.51)	.33	0.87 (0.61 to 1.24)	.44
2	ERBB2-negative	—		—		—		—	
	ERBB2-low	1.04 (0.39 to 2.75)	.94	0.64 (0.28 to 1.48)	.3	0.71 (0.31 to 1.65)	.43	0.76 (0.52 to 1.10)	.14

[1]HR = Hazard Ratio, sHR = Subdistribution Hazard Ratio, CI = Confidence Interval
Model 1 adjusted for age
Model 2 stratified by HR status and adjusted for age, stage, radiation, chemotherapy, hormone therapy, and surgery
Breast Cancer-Specific Mortality assessed using Fine-Gray competing risks models with non-breast cancer-specific mortality as a competing event

© 2024 [First author last name and initials or group name] et al. *[Journal name].*

eTable 7. Associations of a 10% increase in TILs and Breast Cancer Outcomes by ERBB2 and Hormone Receptor Subgroups

Characteristic	Model 1		Model 2	
	HR (95% CI)[1]	p-value	HR (95% CI)[1]	p-value
Overall-Survival				
ERBB2-low	0.96 (0.87 to 1.05)	.34	0.92 (0.83 to 1.02)	.11
ERBB2-negative	1.05 (0.97 to 1.14)	.19	0.98 (0.90 to 1.07)	.7
HR-positive / ERBB2-low	0.96 (0.85 to 1.08)	.49	0.93 (0.82 to 1.05)	.23
HR-negative / ERBB2-low	0.92 (0.76 to 1.10)	.36	0.88 (0.72 to 1.09)	.25
HR-positive / ERBB2-negative	1.07 (0.96 to 1.20)	.22	1.04 (0.92 to 1.17)	.55
HR-negative / ERBB2-negative	0.90 (0.78 to 1.03)	.13	0.93 (0.81 to 1.07)	.32
Recurrence-Free Survival				
ERBB2-low	0.98 (0.89 to 1.09)	.74	0.94 (0.83 to 1.05)	.28
ERBB2-negative	1.05 (0.95 to 1.16)	.31	0.94 (0.84 to 1.05)	.27
HR-positive / ERBB2-low	1.05 (0.94 to 1.18)	.37	1.01 (0.90 to 1.15)	.82
HR-negative / ERBB2-low	0.65 (0.46 to 0.92)	.01	0.59 (0.40 to 0.88)	.009
HR-positive / ERBB2-negative	1.04 (0.90 to 1.20)	.62	0.96 (0.82 to 1.12)	.59
HR-negative / ERBB2-negative	0.89 (0.76 to 1.05)	.17	0.91 (0.78 to 1.07)	.25
Breast Cancer-Specific Mortality				
ERBB2-low	1.03 (0.93 to 1.14)	.58	0.96 (0.83 to 1.12)	.62
ERBB2-negative	1.01 (0.91 to 1.13)	.81	0.95 (0.84 to 1.07)	.38
HR-positive / ERBB2-low	1.09 (0.96 to 1.24)	.19	1.05 (0.90 to 1.21)	.55
HR-negative / ERBB2-low	0.78 (0.57 to 1.06)	.11	0.71 (0.48 to 1.05)	.09
HR-positive / ERBB2-negative	0.97 (0.78 to 1.21)	.78	0.90 (0.71 to 1.16)	.42
HR-negative / ERBB2-negative	0.94 (0.81 to 1.08)	.37	0.96 (0.81 to 1.13)	.6

[1]HR = Hazard Ratio, CI = Confidence Interval
Model 1 adjusted for age at diagnosis,
Model 2 adjusted for age at diagnosis, stage, radiation, chemotherapy, hormone therapy, and surgery. Model 2 in HR-Negative subgroups not adjusted for chemotherapy and hormone therapy

© 2024 [First author last name and initials or group name] et al. *[Journal name].*
