# Supplementary material for: Clinical, Epidemiologic, and Pathologic Significance of ERBB2-Low Expression in Breast Cancer
Source: JAMA Netw Open. 2024 Mar 22;7(3):e243345. doi: 10.1001/jamanetworkopen.2024.3345 (PMC10960203; doi:10.1001/jamanetworkopen.2024.3345)
Supplement: Supplement 2. — Data Sharing Statement [file jamanetwopen-e243345-s002.pdf]

Data Sharing Statement

Khoury. Clinical, Epidemiologic, and Pathologic Significance of ERBB2-Low Expression in Breast Cancer. *JAMA Netw Open*. Published March 22, 2024.
doi:10.1001/jamanetworkopen.2024.3345

Data
Data available: No

Additional Information
Explanation for why data not available: data will be available upon request in a de-identified fashion
